# Proper Authentication of Ancient DNA Is Still Essential

**DOI:** 10.3390/genes9030122

**Published:** 2018-02-26

**Authors:** Raphael Eisenhofer, Laura S. Weyrich

**Affiliations:** Department of Genetics & Evolution, Darling Building, The University of Adelaide, North Terrace, Adelaide, SA 5005, Australia; laura.weyrich@gmail.com

**Keywords:** ancient DNA, microbiome, paleomicrobiology, microbial ecology

Santiago-Rodriguez et al. [1] report on the putative gut microbiome and resistome of Inca and Italian mummies, and find that Italian mummies exhibit higher bacterial diversity compared to the Inca mummies. However, contaminant taxa in their negative control account for most of the biological signal observed. In addition, they fail to properly apply field-standard ancient DNA authentication techniques to their data and self-plagiarize a previously published figure. Poor standards in paleomicrobiological research are currently plaguing the field, despite numerous warnings [2,3,4] and reviews [5,6,7,8] on best practice.

DNA contamination from museums, curators, scientists, soil, and even the laboratory can drive signals present in modern and ancient metagenomics data sets [3,7,9,10,11,12,13,14,15,16]. Therefore, explicit rules and standards to avoid falsely reporting contaminants in metagenomics datasets have been put forth [3,4,6,7,8,17]. These standards typically include sampling and extraction blank controls (e.g., tubes processed without the addition of biological samples) to monitor contaminant DNA and correctly attribute its contribution in subsequent analyses. In the study by Santiago-Rodriquez et al. [1], a non-template or blank control was included in their 16S analysis. However, the authors failed to explore the contaminant species within this control during their analysis of differences between Incan and Italian mummies. We explored the taxa present within their blank control (Supplementary_Dataset_2.txt from their publication) and compared it to those identified within the mummies. We found that laboratory contaminants present within their blank control are driving the differences between Incan and Italian mummies (Figure 1). For example, the five most abundant taxa identified in the Italian mummies (*Sphingomonadales*, *Pseudomonadales*, *Rhizobiales*, *Bacillales*, and *Clostridiales* species) are all found in the blank control. It is also worth noting that these taxa have previously been identified as common laboratory or reagent contaminants in numerous studies [3,14,15]. This strongly suggests that the cultural differences reported by the authors are likely the result of laboratory contamination and calls into question the validity of their subsequent analyses.

The authors then attempt to use MapDamage to assess the authenticity of their shotgun metagenomic ancient DNA; this tool is widely used within the paleomicrobiological field for detecting patterns of cytosine deamination that are characteristic of authentic ancient DNA [18]. Critically, the authors did not provide details as to how they ran the analysis; MapDamage calculates the deamination rate by comparing a reference genome to the mapped target sequences present in a given biological sample (i.e., the reference and target species are typically reported for the analysis). Despite this lack of information, the MapDamage plot provided by the authors in their supplementary information (Figure 2A) is identical to one in a previous publication by the team [4] (Figure 2B), suggesting that the authors self-plagiarized this figure and did not in fact run the analysis.

Despite this, the figure provided also does not support the authenticity of ancient DNA, as the expected C to T at the 5′ and G to A substitutions at 3′ ends of the DNA fragments are not present. The authors defend their lack of authentic ancient DNA signal by stating: “Damage-based ancient DNA authentication tools, such as mapDamage, may be incompatible with ancient microbiome studies unless a high sequencing coverage is reached”. However, simulations and empirical data show that only a few thousand sequences from the genome of interest are required to assess the presence of cytosine deamination [6], and MapDamage has been successfully applied in several paleomicrobiological studies [20,21,22,23]. To investigate if MapDamage could be appropriately applied to the Santiago-Rodriquez et al. data set [1], we downloaded the metagenomic reads from a mummy present within their study (NASD14) and identified species present in the sample using MALT and MEGAN [24,25] against a reference database containing >50-thousand bacterial and archaeal genomes obtained from NCBI Assembly. Similar to the authors’ shotgun results, we identified ≈1.2 million reads assigned to *Sphingomonas* sp. DC-6 (Sphingomonadales), and 33,730 reads assigned to *Vibrio parahaemolyticus* (Vibrionales). We then mapped the metagenomic reads against these reference genomes with the BWA-backtrack (ALN) aligner [26]. The outputs were converted into SAM files then used as input for MapDamage, comparing the “ancient” *Sphingomonas* and *Vibrio* species to their respective reference genome. The resulting plots (Figure 2C,D) clearly illustrate no characteristic ancient DNA damage and are as expected for modern, likely contaminant, DNA. Given that *Sphingomonas* is one of the most abundant taxa in their data and is a known contaminant species, our reanalysis further strengthens the likelihood that contaminant DNA is driving their findings.

To conclude, a reanalysis of Santiago-Rodriquez et al.’s [1] findings strongly suggest that the observed signal is due to laboratory contamination of modern bacterial species. The authors also failed to compare their data to their extraction blanks controls, did not include shotgun metagenomic extraction blanks, and did not authenticate their ancient DNA using MapDamage. Paleomicrobiology is a new and rapidly growing field of research, with little room for plagiarized figures and blatant disregard for best-practice methods. In light of these findings, we suggest either heavy corrections or retraction of the article to prevent further erosion of the scientific integrity of paleomicrobiological research.

## Figures and Tables

**Figure 1:**
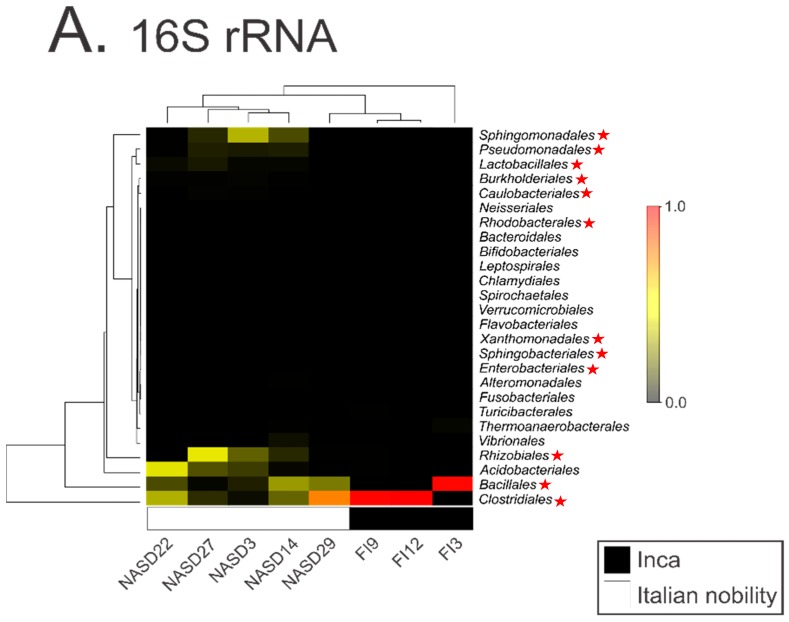
An altered reproduction of Figure 1A from Santiago-Rodriguez et al. [1] where taxa identified in the 16S rRNA blank control are identified in the mummy samples by red stars. The highest-abundance taxa identified in the 16S rRNA data are also present in the 16S rRNA blank control.

**Figure 2:**
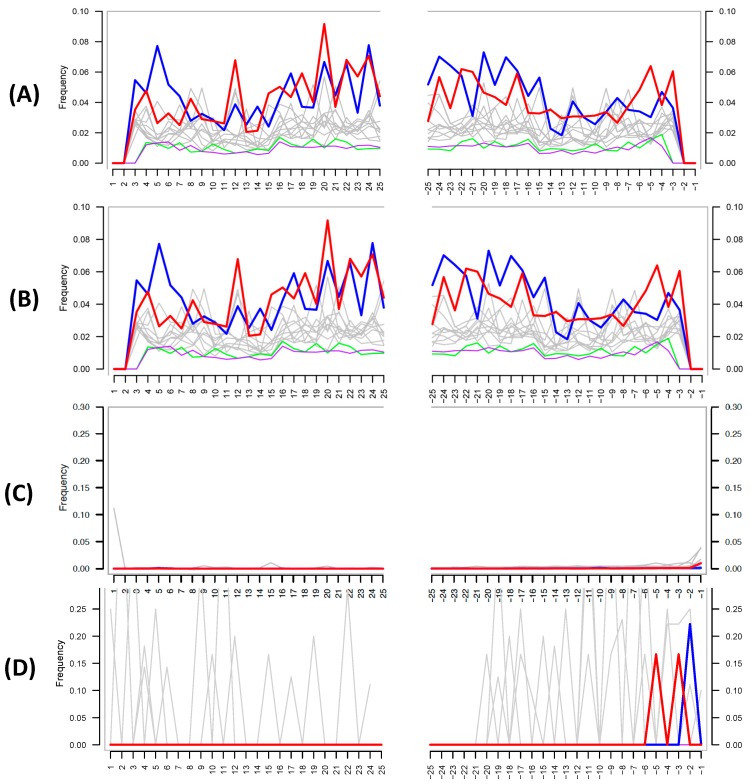
(**A**) MapDamage plot provided by the authors in their latest paper [1]. (**B**) MapDamage plot provided by authors in their previous publication [19]. Both plots are identical, and both show the absence of damage characteristic of authentic ancient DNA. (**C**) MapDamage plot obtained by using reads aligned from Italian mummy NASD14 from Santiago-Rodriquez et al. [1] against the *Sphingomonas sp. DC-6* genome (ASM71517v2). (**D**) Same as (**C**), except using *Vibrio parahaemolyticus* (ASM19609v1), a taxon not found in the authors’ negative control. The lack of nucleotide misincorporation is indicative of modern DNA.
